# Alcohol use, vision therapy, children, mood … what predicts post-traumatic growth after concussion in women?

**DOI:** 10.3389/fgwh.2026.1815889

**Published:** 2026-08-27

**Authors:** Martina Anto-Ocrah, Katherine Kenyon, Celestin Niyomugabo, Michael Chen, Jane Liebschutz, Katherine Snedaker

**Affiliations:** 1 Department of Medicine, Division of General Internal Medicine, University of Pittsburgh School of Medicine, Pittsburgh, PA, United States; 2 Department of Epidemiology, Graduate School of Public Health, University of Pittsburgh, Pittsburgh, PA, United States; 3 Neuroplasticity and Multimodal Imaging (NMI) Lab Cognitive Neuroscience Unit (CNU), Deakin University, Victoria, Australia; 4 VONSUNG, Kigali, Rwanda; 5 Nava PBC, Washington, DC, United States; 6 PINK Concussions, Norwalk, CT, United States

**Keywords:** concussion, females, mild traumatic brain injury, post-traumatic growth, PTSD, women

## Abstract

**Background:**

Post-traumatic growth (PTG) refers to positive psychological change following a traumatic or life-altering event. Concussion, also known as mild traumatic brain injury(mTBI), results from a physical trauma that can lead to psychological outcomes such as post-traumatic stress disorder (PTSD) as well as PTG. Research indicates that PTG and PTSD occur at similar rates among individuals who have experienced concussion, consistent with findings across other trauma-exposed populations. While women often experience more difficult concussion recoveries than men, they often experience greater PTG after trauma. Understanding the factors that support PTG in women with post-concussion symptoms may help guide clinical care to both foster PTG, and identify individuals who may require more targeted recovery support.

**Objectives:**

We examined how demographic, health behaviors, concussion-related, and mental health factors—as well as access to therapeutic services—relate to PTG in women. We also explored the association between PTG and traits such as resilience and flourishing following concussion.

**Methods:**

A cross-sectional survey was conducted from November 10–13, 2023, among U.S.-based women living with persistent post-concussion symptoms. PTG was assessed using the Post-traumatic Growth Inventory (PTGI-X; cutoff: <75 = low PTG, ≥75 = moderate/high PTG), injury details, mood (PHQ-9, GAD-7), and persistent post-concussion symptoms (Rivermead Post-Concussion Questionnaire). Descriptive statistics compared groups. Logistic regression identified associated factors of PTG and estimated corresponding odds ratios.

**Results:**

A total of 434 PINK Concussion members responded to the survey with 116 (27%) completing all questions. The mean PTG score was 70.0 (SD = 26.9; median = 71; range = 1–125). Just over half (51.7%, *n* = 60) had low PTG scores (<75), while 48.3% (*n* = 56) had moderate-to-high PTG scores. High PTG was significantly associated with being African American/Black or Hispanic/Latino (*p* = .007), multiparity (*p* = .013), and having multiple young children at home (*p* = .004). Low PTG participants were more likely to have sought multiple treatments (*p* = .0244), and to have a history of alcohol use (*p* = .025). Vision therapy was more common in the low PTG group (31% vs. 7%, *p* = .001), suggesting it may be linked to unresolved visual symptoms. A higher proportion of high PTG participants had returned to work (66% vs. 45%, *p* = .036). High PTG was also associated with greater resilience (*p* = .005) and flourishing (*p* = .005), with a positive correlation between these traits.

**Conclusion:**

This exploratory study highlights key demographic and psychosocial factors linked to PTG in women recovering from concussion. Modifiable characteristics such as resilience, flourishing, and lifestyle stability, particularly return to work and absence of alcohol use—were associated with higher PTG, offering potential intervention targets and helping to identify individuals who may require more intensive post-concussion support.

## Introduction

According to the Centre for Disease Control and Prevention, between 3.2 million and 5.3 million Traumatic Brain Injuries (TBI) occur in the United States annually (1). An estimated 176 Americans die each day from TBI-related complications (1, 2). Between 80%–90% are classified as mild TBIs (mTBIs) or concussions (3); though “mild” refers to the severity of the trauma, not its consequences. Although many people with mTBI recover well, others develop persistent post-concussion symptoms (PPCS). This is marked by unresolved physical, cognitive, and psychosocial sequelae that span months, even years, after initial injury (4, 5). The morbidity of PPCS is greater in women than it is in men (6, 7), with women reporting worse physical (headaches, dizziness, or fatigue), cognitive (concentration and irritability) and psychosocial (depression, anxiety, and post-traumatic stress disorder (PTSD) symptomatology up to five years post-injury (3, 7–10). Despite this, women remain understudied in the concussion literature.

Researchers often attribute post-trauma deficits to individual-level factors such as race, sexual orientation, familial psychiatric history and amount of social support (11). Emerging research shows, however, that the effects of trauma are not universally negative (11). While some people may experience the deleterious effects of the traumatic event, others may realize psychological benefits. This phenomenon, known as post-traumatic growth (PTG), is defined as the positive, psychological change that results from a struggle through a life-altering experience, which encompasses the following five domains (12–15): improved interpersonal relationships, recognition of new possibilities in one's life course, greater personal strength, spiritual or religious growth, and greater appreciation of life.

Interestingly, research shows the rates of PTG and PTSD (16–18) are similar across various trauma populations, including TBI patients (18, 19); and while women may experience poorer recovery trajectories than men (8), they are more likely to experience PTG (39% of women compared to only 12% of men) and have higher PTG levels than men following a traumatic event (16). Meaning, that while the psychological impact of trauma can be intense for both men and women, women are more likely to derive positive meaning from their experiences, even in the face of recovery challenges. While the literature consistently finds that women experience higher PTG than men, it is still unclear what mechanisms drive this difference. It may be due in part to higher social support coping and empathy being associated with higher PTG, as both factors are reported at greater levels in women than men (20). Similarly, coping behavior, particularly emotion-focused coping, is associated with PTG, and is more highly reported in women than men (20, 21). Moreover, women have been found to ruminate on constructive issues more than men, which has been linked to PTG (20, 22). PTG has been observed in women following traumatic birth (23), intimate partner violence (24, 25), breast cancer (26) and natural disaster (27). Demographic variables such as age, education, socioeconomic status, and race impact trauma recovery (20–24). Moreover, traits such as resilience, defined as the ability to adapt, recover, and grow stronger in the face of challenges, stress, or adversity (25), and greater resilience has been associated with fewer post-concussion symptoms following (26, 27). Other factors influencing PTG include employment (22, 28, 29) spirituality (27, 30, 31), social connection (20, 32, 33), mental health (34, 35), and early access to rehabilitation (20).

As strengths-based approaches-which focus on resilience, recovery, and personal strengths rather than symptom deficits-take the forefront of research (28, 29), it is important to understand the factors that promote PTG as a positive psychosocial outcome that complements traditional measures of recovery. Examining PTG in women with persistent post-concussive symptoms (PPCS), including factors related to whole-person health and biopsychosocial care, may provide additional insight into adaptation, functional recovery, and long-term wellbeing (30, 31). In this exploratory study, we asked: *What modifiable and non-modifiable factors are associated with PTG in women with PPCS?* Specifically, we examined the potential roles of demographic characteristics, health behaviors, concussion history and mental health factors, and access to therapeutic services in PTG. We also assessed the relationships between PTG, resilience, and flourishing to better understand how positive psychological adaptation may manifest following concussion. Findings may generate hypotheses for future personalized rehabilitation approaches.

## Methods

### Participants

We collaborated with PINK Concussions for this research endeavour. PINK Concussions is a free, social-media based non-profit organization whose mission is to develop evidence-based research approaches to support TBI recovery in women and girls (9). Membership is intended for women and girls who have sustained a concussion and are experiencing persistent post-concussive symptoms (PPCS). As part of the membership process, prospective members are pre-screened by PINK Concussions to confirm a history of concussion and the presence of PPCS lasting at least three months before joining the community. Participants in the present exploratory study were recruited exclusively from this membership. Concussion history and PPCS status were therefore established through participant self-report and the organization's membership screening process rather than independent clinician confirmation. The Rivermead Post-Concussion Symptoms Questionnaire (RPQ) was administered to characterize current symptom burden and was not used to determine study eligibility or classify PPCS. Demographic, historic trauma, mood, and concussion-related characteristics were also collected to comprehensively characterize the study sample (Table 1). From November 10–13, 2023, members of the PINK Concussions Facebook group were invited to participate in an online cross-sectional survey via an IRB-approved REDCap survey link (shared in the groups by PINK founder and co-author KS). Upon clicking the link, interested participants were brought to a landing page describing the study and the consent form. To be eligible, participants were required to: (i) be 18 years of age or older; (ii) have been assigned female at birth; (iii) have sustained a concussion and be living with persistent post-concussive symptoms (PPCS), as reflected by membership in the PINK Concussions community; and (iv) reside in the United States. Participants received $40 for their participation.

**Table 1:** Demographic and concussion attributes of study population (*n* = 116).

Age	count	mean	std	min	25%	Median (50%)	75%	**max**
Current age	116	36.9	12.5	20	27	34	46	69

Variable							Frequency	Percentage
**Would you describe yourself as?**								
African American/Black							28	24.1
American Indian/Alaskan Native							2	1.7
Asian							1	0.9
Hispanic/Latino							13	11.2
Multi-racial							1	0.9
White/Caucasian							71	61.2
Total							116	100.0
**What is your romantic relationship status?**								
Single and not in a relationship							18	15.5
In a relationship but not married							39	33.6
Married							54	46.6
Divorced or separated							3	2.6
Missing							2	1.7
Total							116	100.0
**What is your highest level of education completed?**								
High school diploma/GED							10	8.6
Technical training/associate's degree							15	12.9
Some college/bachelor's degree							57	49.1
Master's/advanced/terminal degree							33	28.5
Missing							1	0.9
Total							116	100.0
**What is your average household income?**								
Less than $30,000							17	14.7
$30,000–59,999							31	26.7
$60,000–100,000							40	34.5
More than $100,000							25	21.6
Decline to answer							3	2.6
Total							116	100.0

Concussion Attributes:								
Variable							Frequency	Percentage
**When was your most recent concussion?**								
<1 month ago							7	6.0
1–3 months ago							17	14.7
4–11 months ago							34	29.3
1–2 years ago							19	16.4
3–5 years ago							20	17.2
>5 years ago							18	15.5
Missing							1	0.9
Total							116	100.0
**Was your most recent concussion diagnosed by a medical provider?**								
Yes, it was diagnosed by a medical provider							109	94.0
No, it was self-diagnosed							6	5.2
Missing							1	0.9
Total							116	100.0
**How did you sustain your most recent concussion?**								
Assault							22	18.8
Fall							30	25.6
Motor vehicle accident							24	20.5
Motorcycle accident							5	4.3
Non-fall sports injury							3	2.6
Recreational vehicle accident							3	2.6
Sledding							3	2.6
Struck by motor vehicle							2	1.7
Struck by object							16	13.7
Other mechanism							7	6.0
Missing							1	0.9
Total							116	100.0
**Did you have a history of concussions before your most recent one?**								
Yes							66	56.9
No							46	39.7
Missing							4	3.5
Total							116	100.0
**How many previous concussions did you have before the most recent one?**								
0							1	0.9
1							20	17.2
2							17	14.7
3							8	6.9
4							4	3.5
>=5							14	12.1
Missing							52	44.8
Total							116	100.0

Variable	count	mean	std	min	25%	Median (50%)	75%	max
How many previous concussions did you have before the most recent one?	64	3.0	2.5	0	1	2	4	11
**PPCS and Mood**								
Total Rivermead Score	116	32.8	12.9	1	25	33.5	41	61
PTSD Checklist score	116	30.2	18.2	0	14	30	46.3	78
Total GAD score	116	8.2	5.1	0	4	8	12	21
Total PHQ Score	116	10.3	6.4	0	5	11	15	25

### Survey instruments and questionnaires

#### Measuring PTG

PTG was calculated using the PTG Inventory (PTGI-X) (32), which uses a 6-point Likert scale (0 = not experienced change following injury, 5 = experienced to a great degree following injury). The PTGI-X shows high internal consistency [*α* = .97 (32)] and test-retest reliability (*α* = .71 (33). Scores range from 0 to 125, with greater scores indicating greater growth. There are no standardized cutoff scores for low, moderate, and high PTG. We used scores in the upper range (3–5) as indicative of moderate/high PTG as has been done across the literature (34, 35). Therefore, total PTGI-X scores less than 75 were classified as low, while scores 75 and over were designated moderate/high PTG.

#### Other measures

Participants answered demographic (age, race, marital status, education, etc.), concussion-related (time since injury, mechanism, concussion history, rehabilitation services, etc.) and mood-related questions [PHQ-9 for depression (36, 37), GAD-7 for anxiety (38), PCL for PTSD (39, 40)]. PPCS was evaluated using 16-item Rivermead Post-Concussion Questionnaire (RPQ) (41), which measures somatic, emotional, and cognitive TBI symptoms relative to pre-injury levels. We also measured factors of resilience using the Resilience Scale (42), and flourishing with the flourishing index scale (43). Questions related to “return to work/play/school” were framed around participants' injuries as follows: *Since your injury, have you been able to return to [work/play/school]?* Substance use (alcohol, tobacco, cannabis) questions were framed to assess participants' past and current use as follows: *Which of these best describe you? Response choices were as follows: I currently use tobacco products and I used tobacco products before my injury, I currently use tobacco products but I did not use tobacco products before my injury, I do not currently use tobacco products but I used tobacco products before my injury, I do not currently use tobacco products and did not use tobacco products before my injury.* The survey questions were created collaboratively with, and reviewed by co-author KS (concussion lived experience advisor) for content validity.

### Data analysis

The data analysis was done using Python. Frequency tables were produced, and PTG severity was dichotomized using the PTGI score (cutoff: <75 = low PTG, ≥75 = moderate/high PTG). Dichotomized PTG scores allow us to identify modifiable factors associated with low PTG, which can be used to risk stratify the study population, and identify those at risk of poor trauma recovery outcomes, giving patients and their caregivers targeted goals for rehabilitation. Shapiro–Wilk tests were first conducted to assess normality of the PTG score distribution and of other continuous variables. Where normality was met, independent-samples *t*-tests were used to compare continuous variables between PTG groups; where violated, the Mann–Whitney *U*-test was used instead. Fisher's exact test or the Chi-square test (as appropriate given expected cell counts) was used to compare categorical variables between the moderate-high and low PTG groups across the different demographic, mood-based and injury-related variables. We used a significance level of *p* < .2 to be conservative, given this is the first exploration of PTG outcomes in women with concussion that we are aware of. Crude and adjusted logistic regression analyses were performed to estimate the association between each predictor and the outcome of interest. Crude (bivariate) logistic regression models were first fit for each candidate predictor, and variables with a significance level of *p* < 0.05 were retained for inclusion in the adjusted multivariable model. The final adjusted model included return-to-work status, current alcohol consumption, race, number of concussion-related services received, and vision therapy status. Odds ratios (ORs) with 95% confidence intervals (CIs) are reported for both the crude and adjusted models. Model fit was evaluated using the pseudo *R*², Akaike information criterion (AIC), Bayesian information criterion (BIC), log-likelihood, and likelihood ratio (LR) test *p*-value for each logistic regression model. Multicollinearity among predictors in the adjusted model was assessed using variance inflation factors (VIFs), with all VIF values ranging from 1.10 to 2.08, indicating no evidence of problematic multicollinearity. For continuous outcome comparisons between groups, normality was assessed using the Shapiro–Wilk test. Where normality was violated, the non-parametric Mann–Whitney *U*-test was used in place of the independent-samples *t*-test. For OLS models, multicollinearity (Condition Number, VIF) were reviewed.

#### Missing data

Analyses were conducted using complete-case data after data cleaning; therefore, sample sizes varied slightly across models because of missing data for specific predictors. Sample sizes are noted for each model as appropriate.

#### PTG domains

We standardized the five PTG domains (relating to others, new possibilities, personal strength, spiritual and existential change, appreciation of life) to have a fixed score of 35 rather than averaging values at different scores for each scale to identify differences between domains. This standardization was necessary because each domain has a different number of questions which affect the total scores for each. Standardization allows for equal comparisons across domains as averaging the value at different scores would be misleading. Following checks for significance using a one-way ANOVA, we administered Tukey's HSD test to determine which domains differed, using a family-wise error rate (FWER) of 0.05 to control for multiple comparisons.

## Results

**Figure 1:**
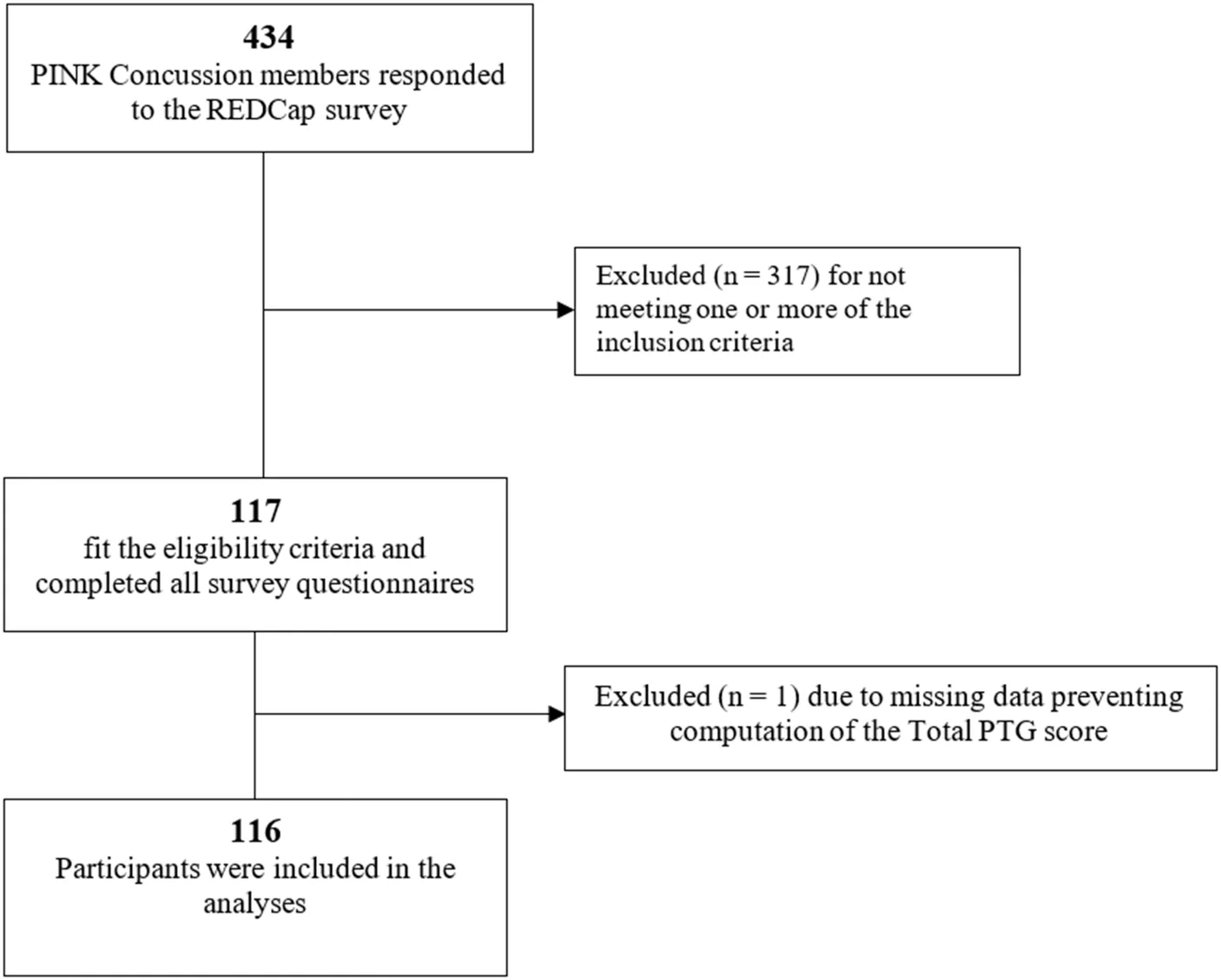
Flow chart of study participants.

From November 10–13, 2023, 434 PINK Concussion members responded to the REDCap survey, with a total of 117 fitting the eligibility criteria and completing all survey questionnaires. The PTG score was not computed for one participant with missing data, and they were excluded from the analysis. Thus, the final analyses included 116 of the 434 participants (27%). Figure 1.

**Figure 2:**
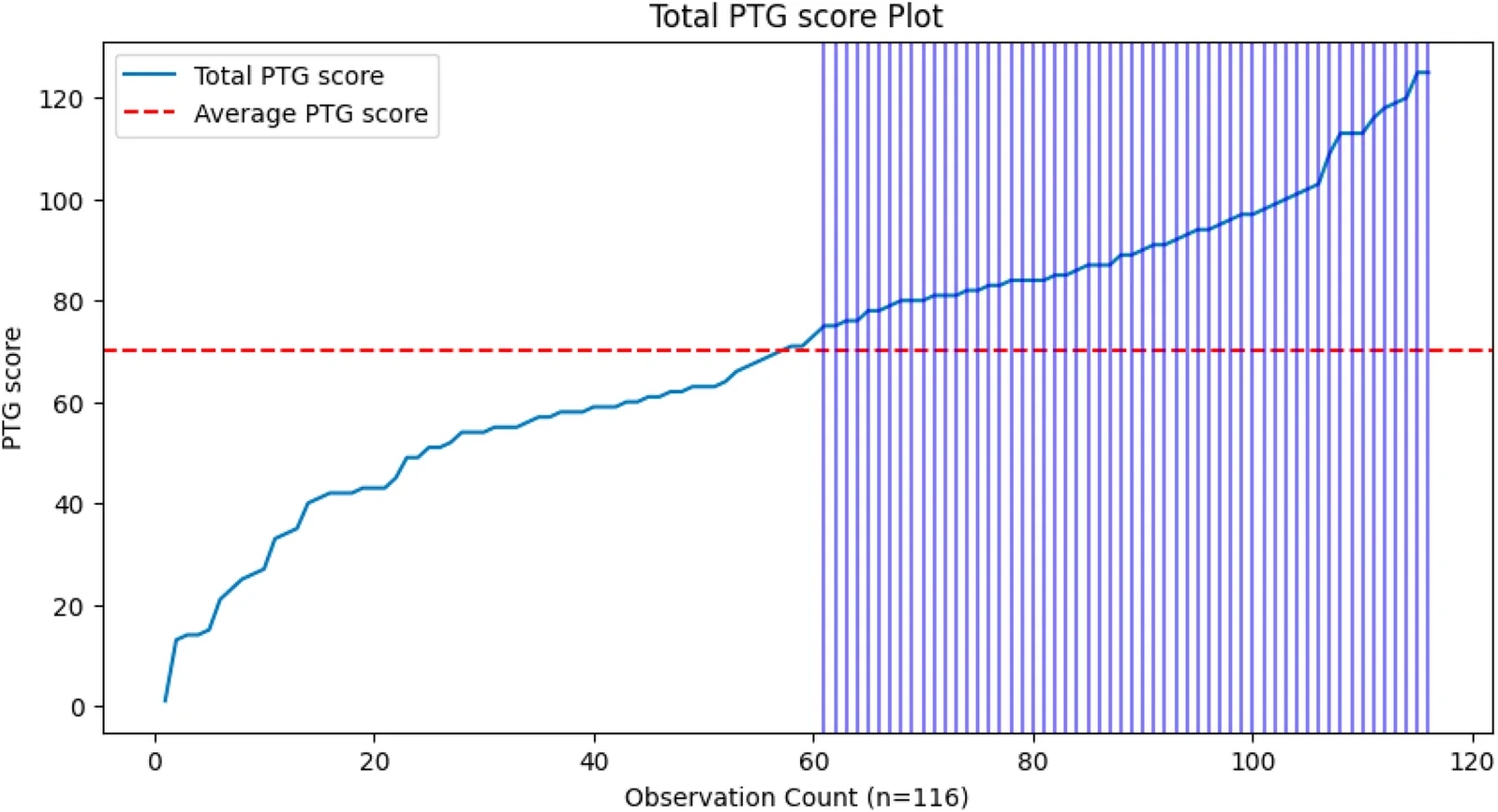
Distribution of PTG scores across the study population.

The Shapiro–Wilk test found no evidence of our data violating the normality assumption (*p* = .354). The mean PTG score for the cohort was 70.0 (±26.85 std; median = 71, range 1–125), with 51.7% (*n* = 60) having a low PTG score (below 75), and 48.3% (*n* = 56) having a moderate/high PTG score (PTGI-X ≥ 75; See Figure 2).

Table 1 provides demographic, historic trauma, mood, and concussion-related characteristics that describe the study population. Participants ranged from 20 to 69 years of age (mean age 36.9 ± 12.5 STD). The majority self-described as White (61%) and about a quarter were African American/Black (24%). 11% were of Hispanic/Latino ethnicity. 94% of participants had clinically diagnosed concussion, and the mean RPQ scores was 32.85 (±12.95 std); range 1–61. An RPQ cutoff of ≥16 is often used as the threshold for significant PPCS burden (41, 44) So the participants in this study had a high burden of post-concussion symptoms.

**Figure 3:**
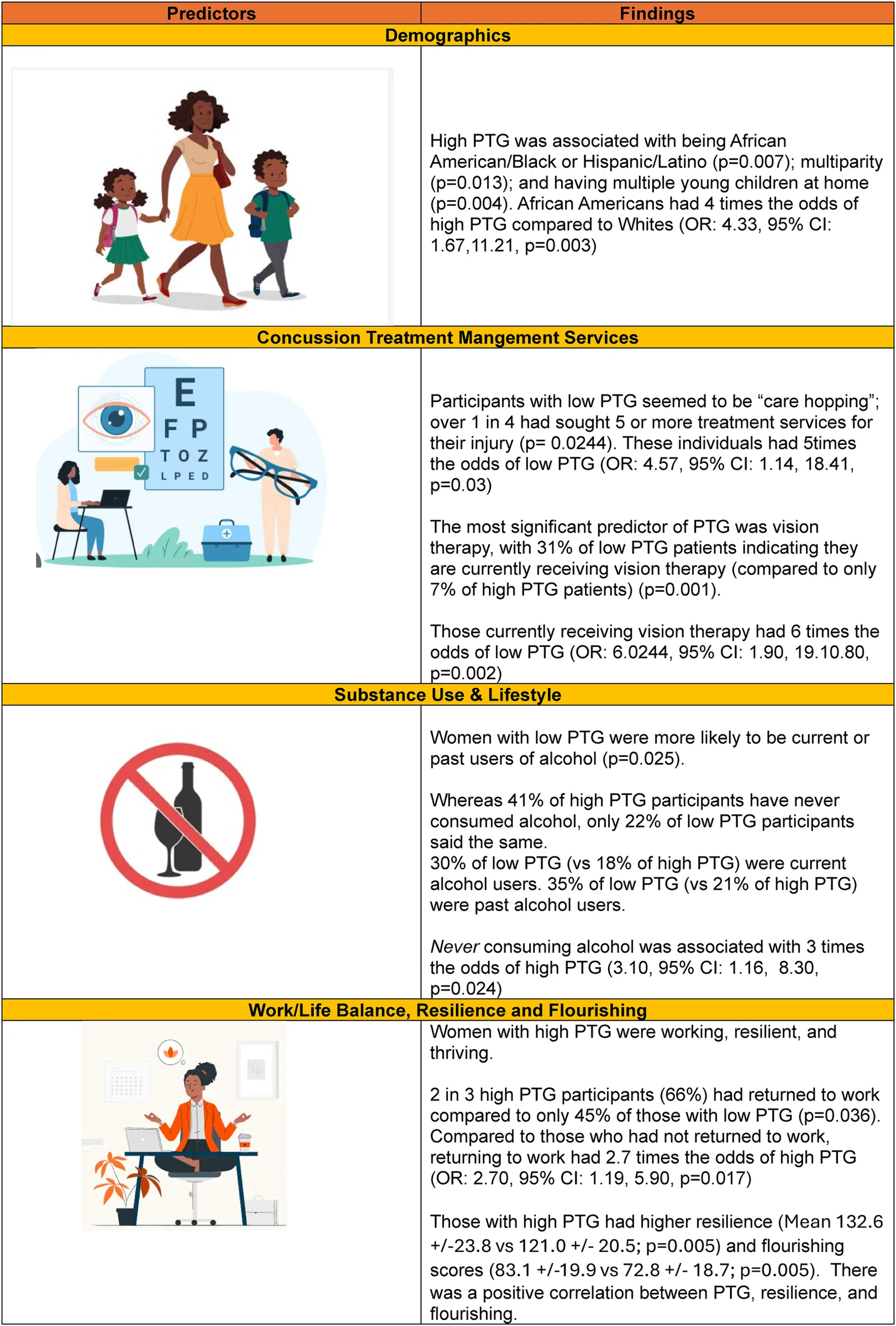
Schematic of findings (See Supplemental files for additional data).

Of the demographic variables (Table 2, Figure 3), only race and number of children differed significantly between low and moderate/high PTG scoring participants, with African American/Black and Hispanic/Latino participants (*p* = .007), those with high parity (*t* = −2.5, *p* = .013) and participants with more young children living in their households (*t* = −3.0, *p* = .004) having higher levels of PTG. Interestingly, neither RPQ nor mood (anxiety, depression and PTSD) was significantly associated with PTG level.

**Table 2:** Demographic and psycho-social factors associated with PTG.

A) Within group comparison of race/ethnicity					
	African American/Black	Hispanic/Latino	Caucasian	Other	Total
Low	8 (28.6%)	5 (38.5%)	45 (63.4%)	2 (50%)	60 (51.7%)
Moderate-high	20 (71.4%)	8 (61.5%)	26 (36.6%)	2 (50%)	56 (48.3%)
Total	28 (100%)	13 (100%)	71 (100%)	4 (100%)	116 (100%)
**F-statistic: 4.28, *P*-value:.007**					

B) Summary Statistics						
	N	Mean (SD)	Min	Max	T	*P*-value
**What is your current age? (Cohen's d: 0.30)**						
Low	60	38.7 (12.7)	21	68	1.6	0.107
Moderate to high	56	35.0 (12.1)	20	69		
Total	116	36.92 (12.51)	20	69		
**How many children have you had? (Cohen's d: −0.47)**						
Low	58	0.8 (1.1)	0	3	−2.5	0.013
Moderate to high	56	1.4 (1.3)	0	5		
Total	114	1.1 (1.2)	0	5		
**How many living children under the age of 18 are in your household? (Cohen's d: −0.56)**						
Low	58	0.6 (0.7)	0	2	−3.0	0.004
Moderate to high	56	1.0 (0.9)	0	3		
Total	114	0.8 (0.8)	0	3		
**PHQ-9 score (Cohen's d: 0.29)**						
Low	60	11.2 (5.9)	1	25	1.6	0.116
Moderate to high	56	9.4 (6.9)	0	22		
Total	116	10.3 (6.4)	0	25		
**GAD-7 score (Cohen's d: 0.24)**						
Low	60	8.8 (4.8)	0	21	1.3	0.206
Moderate to high	56	7.6 (5.5)	0	21		
Total	116	8.2 (5.14)	0			
**PCL score (Cohen's d: −0.07)**						
Low	60	29.6 (17.3)	0	68	-0.4	0.719
Moderate to high	56	30.8 (19.2)	2	78		
Total	116	30.2 (18.2)	0	78		
**RPQ score (Cohen's d: 0.1)**						
Low	60	33.5 (13.1)	1	61	0.5	0.61
Moderate to high	56	32.2 (12.9)	3	59		
Total	116	32.9 (13.0)	1	61		

Regarding concussion-related factors (Table 3, Figure 2), the groups did not differ significantly on any indicators of injury severity, including time since injury (*p* = .33), injury mechanism (*p* = .15), or history of prior concussions (*p* = .47). However, service utilization post-concussion was significantly associated with PTG. Over a quarter of low PTG participants indicated that they have accessed 5 or more services since their concussion (*p* = .024). When asked about the types of concussion management service participants received, only vision therapy showed statistical significance, with over 30% of low PTG participants indicating that they were currently receiving vision therapy treatment (*p* = .001). None of the other commonly utilized concussion management services (chiropractic, neurology, endocrine, social work, mental health, etc) were statistically significant and did not show a trend in either. Insurance coverage was about the same between the groups, with over 94% indicating that they were insured (*p* = 1.0). Therefore, service access was not limited by insurance coverage for the majority of study participants.

**Table 3:** Concussion-related factors associated with PTG.

**Variables/levels**	**Low PTG**	**Moderate to high PTG**	**Total**	***P*-value**
**When was your most recent concussion? (Cramer's V: 0.225)**				**Fisher *p*-value**
<1 month ago	3 (5%)	4 (7.1%)	7 (6%)	0.3328
1–3 months ago	6 (10%)	11 (19.6%)	17 (14.7%)	
4–11 months ago	19 (31.7%)	15 (26.8%)	34 (29.3%)	
1–2 years ago	8 (13.3%)	11 (19.6%)	19 (16.4%)	
3–5 years ago	14 (23.3%)	6 (10.7%)	20 (17.2%)	
>5 years ago	10 (16.7%)	8 (14.3%)	18 (15.5%)	
Missing	0 (0%)	1 (1.8%)	1 (0.9%)	
Total	60 (100%)	56 (100%)	116 (100%)	
**How did you sustain your most recent concussion? (Cramer's V: 0.24)**				**Chi2 *p*-value**
Assault	7 (11.7%)	15 (26.8%)	22 (19%)	0.153
Fall	14 (23.3%)	16 (28.6%)	30 (25.9%)	
Motor vehicle/Motorcycle accident	16 (26.7%)	13 (23.2%)	29 (25%)	
Struck by object/motor vehicle	12 (20%)	6 (10.7%)	18 (15.5%)	
Other	11 (18.3%)	6 (10.7%)	17 (14.7%)	
Total	60 (100%)	56 (100%)	116 (100%)	
**Did you have a history of concussions before your most recent one? (Cramer's V: 0.067)**				**Chi2 *p*-value**
Yes	33 (55%)	33 (58.9%)	66 (56.9%)	0.474
No	27 (45%)	19 (33.9%)	46 (39.7%)	
Missing	0 (0%)	4 (7.1%)	4 (3.4%)	
Total	60 (100%)	56 (100%)	116 (100%)	
**How many services did you receive due to your concussion/mTBI? (Cramer's V: 0.285)**				**Fisher *p*-value**
0	7 (11.7%)	10 (17.9%)	17 (14.7%)	0.0244
1–2	30 (50%)	26 (46.4%)	56 (48.3%)	
3–4	7 (11.7%)	15 (26.8%)	22 (19%)	
5+	16 (26.7%)	5 (8.9%)	21 (18.1%)	
Total	60 (100%)	56 (100%)	116 (100%)	
**Are you receiving chiropractic therapy due to your concussion/mTBI? (Cramer's V: 0.042)**				**Chi2 *p*-value**
Yes	11 (18.3%)	13 (23.2%)	24 (20.7%)	0.675
No	49 (81.7%)	43 (76.8%)	92 (79.3%)	
Total	60 (100%)	56 (100%)	116 (100%)	
**Are you receiving counseling service due to your concussion/mTBI? (Cramer's V: 0.051)**				**Chi2 *p*-value**
Yes	27 (45%)	29 (51.8%)	56 (48.3%)	0.586
No	33 (55%)	27 (48.2%)	60 (51.7%)	
Total	60 (100%)	56 (100%)	116 (100%)	
**Are you receiving endocrine/hormonal therapy due to your concussion/mTBI? (Cramer's V: 0.078)**				**Fisher *p*-value**
Yes	4 (6.7%)	1 (1.8%)	5 (4.3%)	0.3655
No	56 (93.3%)	55 (98.2%)	111 (95.7%)	
Total	60 (100%)	56 (100%)	116 (100%)	
**Are you receiving neurology/neuro service due to your concussion/mTBI? (Cramer's V: 0.088)**				**Chi2 *p*-value**
Yes	22 (36.7%)	15 (26.8%)	37 (31.9%)	0.346
No	38 (63.3%)	41 (73.2%)	79 (68.1%)	
Total	60 (100%)	56 (100%)	116 (100%)	
**Are you receiving occupational therapy (OT) service due to your concussion/mTBI? (Cramer's V: 0)**				**Chi2 *p*-value**
Yes	12 (20%)	11 (19.6%)	23 (19.8%)	1
No	48 (80%)	45 (80.4%)	93 (80.2%)	
Total	60 (100%)	56 (100%)	116 (100%)	
**Are you receiving physical therapy service due to your concussion/mTBI? (Cramer's V: 0.029)**				**Chi2 *p*-value**
Yes	21 (35%)	17 (30.4%)	38 (32.8%)	0.738
No	39 (65%)	39 (69.6%)	78 (67.2%)	
Total	60 (100%)	56 (100%)	116 (100%)	
**Are you receiving psychiatry service due to your concussion/mTBI? (Cramer's V: 0.083)**				**Chi2 *p*-value**
Yes	11 (18.3%)	6 (10.7%)	17 (14.7%)	0.37
No	49 (81.7%)	50 (89.3%)	99 (85.3%)	
Total	60 (100%)	56 (100%)	116 (100%)	
**Are you receiving vision therapy service due to your concussion/mTBI? (Cramer's V: 0.286)**				**Fisher *p*-value**
Yes	19 (31.7%)	4 (7.1%)	23 (19.8%)	0.001
No	41 (68.3%)	52 (92.9%)	93 (80.2%)	
Total	60 (100%)	56 (100%)	116 (100%)	
**Are you not receiving any service due to your concussion/mTBI? (Cramer's V: 0.066)**				**Chi2 *p*-value**
Yes	7 (11.7%)	10 (17.9%)	17 (14.7%)	0.497
No	53 (88.3%)	46 (82.1%)	99 (85.3%)	
Total	60 (100%)	56 (100%)	116 (100%)	
**Do you currently have health insurance coverage? (Cramer's V: 0)**				**Fisher *p*-value**
Yes	56 (93.3%)	53 (94.6%)	109 (94%)	1
No	4 (6.7%)	3 (5.4%)	7 (6%)	
Total	60 (100%)	56 (100%)	116 (100%)	

With regards to health behaviors (Table 4, Supplementary Figure S2), 41% of participants with high PTG reported never consuming alcohol, in contrast to just 22% of those with low PTG. Among women with low PTG, there was a higher prevalence of a history of alcohol use. Specifically, 35% of these women, compared to only 21% in the high PTG group, reported past alcohol consumption before their injury but abstain now. Furthermore, 30% of women in the low PTG group, compared to 18% in the high PTG group, identified as both current and past alcohol users (*p* = 0.025). This suggests that pre-injury history of alcohol use may play a role in the differing PTG outcomes, possibly through neurobiological or behavioral processes linked to addiction. Nevertheless, alcohol use may also reflect broader pre-existing lifestyle or psychosocial factors, and future studies distinguishing pre- and post-injury alcohol use are needed to better understand these relationships. There were no associations found for tobacco or marijuana (Supplementary).

**Table 4:** Health behavioral factors associated with PTG & work/life outcomes.

**Variables/levels**	**Low PTG**	**Moderate to high PTG**	**Total**	***P*-value**
**Which of these best describe you? (Cramer's V: 0.218)**				**Fisher *p*-value**
I currently use tobacco products and I used tobacco products before my injury	3 (5%)	9 (16.1%)	12 (10.3%)	0.1618
I currently use tobacco products but I did not use tobacco products before my injury	6 (10%)	2 (3.6%)	8 (6.9%)	
I do not currently use tobacco products but I used tobacco products before my injury	10 (16.7%)	11 (19.6%)	21 (18.1%)	
I do not currently use tobacco products and did not use tobacco products before my injury	39 (65%)	34 (60.7%)	73 (62.9%)	
Missing	2 (3.3%)	0 (0%)	2 (1.7%)	
Total	60 (100%)	56 (100%)	116 (100%)	
**Which of these best describe you? (Cramer's V: 0.292)**				**Fisher *p*-value**
I currently consume alcohol and I used to consume alcohol before my injury	18 (30%)	10 (17.9%)	28 (24.1%)	0.0249
I currently consume alcohol but I did not consume alcohol before my injury	4 (6.7%)	9 (16.1%)	13 (11.2%)	
I do not currently consume alcohol but I used to consume alcohol before my injury	21 (35%)	12 (21.4%)	33 (28.4%)	
I do not currently consume alcohol and did not consume alcohol before my injury	13 (21.7%)	23 (41.1%)	36 (31%)	
Missing	4 (6.7%)	2 (3.6%)	6 (5.2%)	
Total	60 (100%)	56 (100%)	116 (100%)	
**Which of these best describe you? (Cramer's V: 0.167)**				**Fisher *p*-value**
I currently use marijuana and I used marijuana before my concussion	11 (18.3%)	6 (10.7%)	17 (14.7%)	0.4216
I currently use marijuana but I did not use marijuana before my concussion	7 (11.7%)	6 (10.7%)	13 (11.2%)	
I do not currently use marijuana but I used marijuana before my concussion	6 (10%)	2 (3.6%)	8 (6.9%)	
I do not currently use marijuana and did not use marijuana before my concussion	34 (56.7%)	36 (64.3%)	70 (60.3%)	
Missing	2 (3.3%)	6 (10.7%)	8 (6.9%)	
Total	60 (100%)	56 (100%)	116 (100%)	
**Since your injury, have you been able to return to play? (Cramer's V: 0.087)**				**Chi2 *p*-value**
Yes	22 (36.7%)	28 (50%)	50 (43.1%)	0.348
No	23 (38.3%)	19 (33.9%)	42 (36.2%)	
Not Applicable	14 (23.3%)	9 (16.1%)	23 (19.8%)	
Missing	1 (1.7%)	0 (0%)	1 (0.9%)	
Total	60 (100%)	56 (100%)	116 (100%)	
**Since your injury, have you been able to return to school/academics? (Cramer's V: 0.123)**				**Chi2 *p*-value**
Yes	20 (33.3%)	26 (46.4%)	46 (39.7%)	0.336
No	16 (26.7%)	11 (19.6%)	27 (23.3%)	
Not Applicable	23 (38.3%)	18 (32.1%)	41 (35.3%)	
Missing	1 (1.7%)	1 (1.8%)	2 (1.7%)	
Total	60 (100%)	56 (100%)	116 (100%)	
**Since your injury, have you been able to return to work? (Cramer's V: 0.215)**				**Chi2 *p*-value**
Yes	27 (45%)	37 (66.1%)	64 (55.2%)	0.036
No	29 (48.3%)	15 (26.8%)	44 (37.9%)	
Not Applicable	2 (3.3%)	4 (7.1%)	6 (5.2%)	
Missing	2 (3.3%)	0 (0%)	2 (1.7%)	
Total	60 (100%)	56 (100%)	116 (100%)	

**Figure 4:**
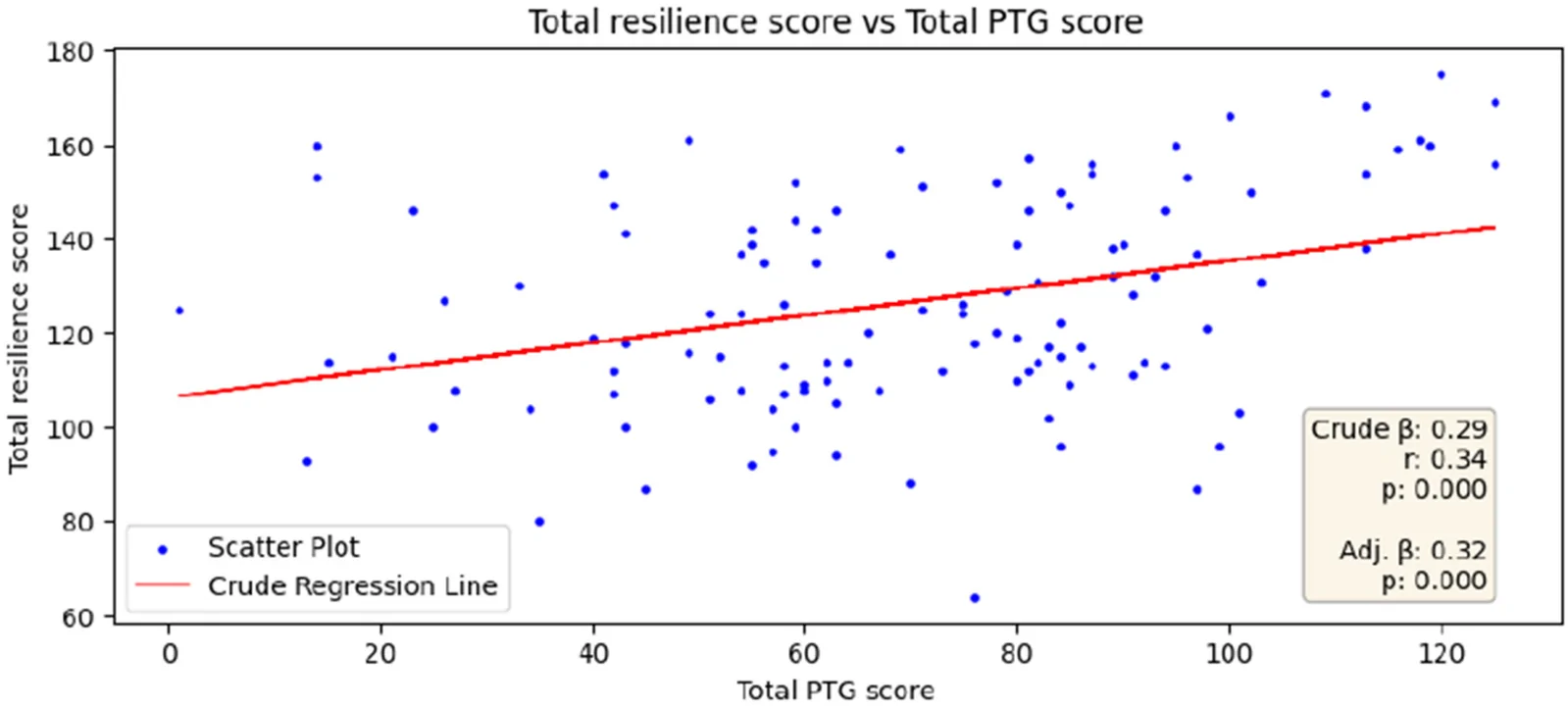
Correlation between PTG scores and total flourishing scores. *Adjusted for race/ethnicity.

Two-thirds of moderate-high PTG participants (66%) had been able to return to work compared to only 45% of those with low PTG (Table 4, Supplementary Figure S3). Thus, PTG could potentially play a meaningful role in participants' earning potential and their overall wellbeing; a finding that was reflected in our analyses of flourishing and resilience (Figures 3, 4).

There were statistically significant associations between PTG, resilience, and flourishing. Participants with high PTG had higher resilience scores than those with low PTG (Mean 132.6 ± 23.8 vs. 121.0 ± 20.5; *p* = .005, Supplementary) and had higher flourishing scores (mean 83.1 ± 19.9 vs. 72.8 ± 18.7; *p* = .005, Supplementary). There was a positive correlation between PTG, resilience, and flourishing. Every unit increase in PTG increased resilience scores by 0.32 points (Figure 3) and flourishing by 0.28 points (Figure 4). All were statistically significant.

### PTG domains

Our analyses revealed significant differences in the mean scores across the five PTG domains (Table 5: *F* = 8.530, *p* *<* *0.001*). The domain with the highest standardized mean was “Appreciation of Life” (Standardized mean = 23.5747), while “Spiritual and Existential Change” had the lowest average (**S**tandardized mean = 17.1884), suggesting that the domains of PTG are not uniformly experienced by all. Further shown in Table 5, the Tukey's HSD test indicated that scores for the “Appreciation of life” domain were significantly different from the scores of all the other domains, but the other domains did not significantly differ from each other. Thus, “Appreciation of Life” is the most pronounced PTG attribute for these TBI survivors.

**Table 5:** PTG domain analyses using tukey's HSD test for multiple comparison of means.

Domains	Standardized mean
Relating to Others	20.1293
New Possibilities	19.0207
Personal Strength	20.0496
Spiritual and Existential Change	17.1884
Appreciation of Life	23.5747
**F-statistic: 8.530; *p*-value: 1.09 × 10^−06^**	

Domain 1	Domain 2	Mean diff	Lower	Upper	Reject
Relating to others	New possibilities	−1.1086	−4.192	1.9751	False
Relating to others	Personal strength	−0.0797	−3.163	3.004	False
Relating to others	Spiritual/existential change	−2.9409	−6.025	0.1427	False
Relating to others	Appreciation of life	3.4454	0.3617	6.5291	*True*
New possibilities	Personal strength	1.0289	−2.055	4.1126	False
New possibilities	Spiritual/existential change	−1.8323	−4.916	1.2514	False
New possibilities	Appreciation of life	4.5541	1.4704	7.6377	*True*
Personal strength	Spiritual/existential change	−2.8612	−5.945	0.2225	False
Personal strength	Appreciation of life	3.5252	0.4415	6.6089	*True*
Spiritual/existential change	Appreciation of life	6.3864	3.3027	9.4701	*True*

## Discussion

This exploratory study aimed to examine the factors that influence post traumatic growth (PTG) in female patients with concussions. We investigated the potential impact of demographic and health behavior characteristics, concussion-related and mental health variables, and access to therapeutic services on PTG. Additionally, we explored the associations between PTG and individual traits such as spirituality, resilience and flourishing to gain a deeper understanding of how PTG manifests in women following concussion/mTBI. In line with previous research (45–49), African American/Black and Hispanic/Latino participants, reported higher levels of PTG following concussion compared to Caucasian participants, who were more likely to have low PTG. This finding can be understood through several interrelated psychological, social, and cultural factors. McLaughlin and colleagues (50) note that minority groups who routinely encounter discrimination, stigma and oppression in the United States often have greater psychological preparedness to experience traumatic events. These experiences might prepare individuals to process new traumas more *adaptively,* leading to growth. These minoritized groups often have shared historical narratives of survival, resistance, and strength (e.g., the Civil Rights Movement, Indigenous resilience, immigration struggles), which often lead to the creation of tight-knit support systems that promote collectivism, interdependence, and family/community bonds. Narratives fostered by these shared, collective experiences may foster an *identity of strength* and encourage growth after trauma as part of a cultural/historical legacy. If the trauma is perceived as unjust or rooted in systemic oppression, it may fuel a desire for positive transformation—both personally and socially, which can lead to greater personal growth, redefined priorities, or renewed purpose—all key dimensions of PTG (51–54).

PTG is also closely linked to how individuals make meaning of suffering. Acquired brain injury survivors often experience a change in perspective and reorganization of life priorities, finding new meaning and purpose (55, 56). Many minority cultures place a strong emphasis on spiritual beliefs, religion, or philosophical frameworks that help contextualize trauma and promote growth (e.g., “suffering strengthens the soul” or “everything happens for a reason”). This can aid in finding purpose and meaning, a core mechanism of PTG. These findings are in line with our earlier work on resilience and concussion recovery (49) in a separate cohort of female concussion patients, which showed that compared with Caucasians, minority individuals with higher resilience reported greater resolution of their PPCS post-injury. Even though race is a non-modifiable social construct, these lines of work point to how critical it is to consider the protective roles of positive psychological factors in research involving women's concussion recovery. Focusing solely on deficit outcomes of disease may not provide the complete recovery picture. In fact, this was supported by our analyses. We did not find any relationship between PTG and mental health or concussion-related factors; including PPCS, which we would have expected to be a statistically significant factor. This may be due to the heterogeneity of post-traumatic responses. Studies have found varying relationships between post-traumatic stress, depression and anxiety symptoms, and PTG in women (26, 27, 57). Further, Pais-Hrit and colleagues (58) found no relationship between PTGI scores and mental health conditions or PTGI and post-injury disability measured by the Glasgow Outcome Scale. These findings support the NIH's assertion that a *whole person approach* that acknowledges *health as the interconnectedness of physical, mental, social, and spiritual factors-not just the absence of disease-*is crucial to understanding and promoting patients' wellbeing and recovery (30, 31).

One of the modifiable factors associated with PTG was access to concussion treatment and management services. Our findings showed that participants with low PTG accessed a greater number of concussion-related services than those who reported higher PTG scores. Interestingly, out of all services sought, only vision therapy was significantly associated with PTG. None of the “standard”, first line concussion treatments—neurology, physical & occupational therapy, psychiatry/mental health services were significantly associated with the outcome. This finding is in line with the literature on mechanistic pathways of poor concussion recovery and adds to the emerging field of neuro-ophthalmology. Visual symptoms can arise following mTBI without any injury to the eyes themselves, at times unbeknownst to the patient. Studies show that over 80 (59–64). Our findings that vision therapy is associated with PTG aligns with this body of work and provides impetus for recommending vision therapy for concussion symptom management.

However, it should be noted that our findings depict an exploratory association and do not suggest that vision therapy directly promotes PTG or that PTG reflects symptom improvement. Rather, the association may reflect the broader context of engaging in targeted rehabilitation, developing coping strategies, or adapting to challenges following concussion. We acknowledge that greater symptom burden may contribute to increased healthcare utilization and may partially explain the observed association between service use such as vision therapy and PTG. However, these findings may reflect the coexistence of ongoing challenges and positive psychological adaptation. Experiencing persistent symptoms and engaging in healthcare services does not preclude the development of PTG. In fact, accessing supportive and rehabilitative services may provide opportunities for education, coping, self-efficacy, and adaptation that contribute to positive change following concussion. As strengths-based approaches—which emphasize resilience, recovery, and personal strengths rather than symptom deficits—continue to inform concussion research, it is important to consider PTG as a complementary psychosocial outcome that can occur alongside symptom burden and ongoing treatment needs. Therefore, future longitudinal studies are needed to better understand the temporal relationship among symptom burden, healthcare utilization, and PTG. Vision-based interventions may be beneficial for individuals with PPCS and represent a potentially valuable treatment approach, but further research is required to better characterize the relationship between vision therapy exposure and clinical outcomes, including the effects of treatment duration, adherence, specific therapeutic approaches, and timing of intervention.

Another modifiable factor associated with PTG was alcohol consumption. Whereas participants with low PTG were more likely to have a history of alcohol consumption, high PTG was associated with *no* history of alcohol consumption. The relationship between TBI and alcohol consumption is complex, with emerging research indicating that alcohol use may play a significant role in shaping concussion recovery outcomes. Studies have shown that alcohol consumption, particularly in the context of post-TBI recovery, can hinder cognitive function, delay healing, and exacerbate symptoms like mood instability, fatigue, and impaired decision-making, which in turn can negatively impact PTG (65, 66). In contrast, high levels of PTG have been consistently associated with individuals who abstain from alcohol (67, 68), suggesting that refraining from alcohol consumption may facilitate more positive psychological adaptation and greater resilience following trauma. Participants with low PTG scores are more likely to be past or current users of alcohol, which may be linked to self-medication behaviors to cope with the psychological and physical challenges that accompany trauma. Alcohol use in these cases may serve as a short-term means of managing pain, sleep disturbances, or emotional distress (69) but can ultimately hinder the brain's healing process and the individual's ability to achieve meaningful growth. As research continues to explore the intersection of TBI, alcohol use, and PTG, it is becoming increasingly clear that abstinence or moderation in alcohol consumption can be an important factor in fostering recovery and facilitating post-traumatic transformation. As shown in the literature, interventions targeted at the first 6 months post-injury may be the most effective for achieving this goal (69). The current study cannot determine whether alcohol use influences the development of PTG or instead reflects differences in psychological adaptation or other pre-existing lifestyle factors. Future research distinguishing pre- and post-injury alcohol use is needed to better understand these relationships.

Women who experienced high levels of PTG tended to be multiparous, with several young children living in their households. Research by Powell et al. (32) found that a strong sense of purpose or personal meaning was the most significant predictor of PTG, and for many women, that sense of purpose is deeply rooted in motherhood. The role of a mother provides a powerful source of meaning and resilience (51), especially for those navigating life after TBI. This dynamic is particularly pronounced among African American women, the subgroup in our study that showed the highest levels of PTG (48). Motherhood in this context appears to serve as both a grounding force and a catalyst for growth, helping women to not only cope with the challenges of TBI but to find strength and a renewed sense of purpose in their children. *Although parity is a modifiable factor, women cannot be forced to reproduce.* However, it is important to recognize that the nurturing responsibilities and emotional bonds tied to raising young children can create a unique resilience, reinforcing the idea that motherhood is not just a life role, but a core element of personal recovery and transformation following trauma.

Finally, those with high PTG also reported greater resilience and a stronger sense of flourishing. A key contributor to these outcomes could be engagement in work. A greater proportion of women with high PTG had returned to work, compared to those with low PTG. Employment not only provides financial stability but also serves as a powerful tool for psychological and emotional recovery following trauma. For many individuals, returning to work or maintaining employment after experiencing a concussion represents a significant milestone, symbolizing not only physical recovery but also the restoration of self-worth and purpose (70). This sense of purpose is crucial for PTG, as it enables individuals to transcend the trauma and rebuild a sense of meaning and an appreciation of life. Pais-Hrit and colleagues (58) found that people who were able to return to work following TBI scored higher on the Changes in Outlook Questionnaire's Positive subscale, another measure of PTG (58). The benefits of employment in the context of PTG are particularly pronounced in women, who often experience multiple roles as caregivers, workers, and community members. Working can provide a sense of balance and integration, helping women maintain their roles in the family and society while also facilitating personal growth. Thus, for women recovering from TBI, employment is not just a source of income, but a vital pathway to psychological recovery, greater well-being, and long-term flourishing. People are more likely to return to work following brain injury if they feel they have strong social support (71). Perceived social support also has been linked to higher resilience and PTG following brain injury (72–74) While we did not include social support as a factor in our analysis, we note that the present study's participants were drawn from an online social support group.

In the spirit of the “appreciation of life” reflected in PTG within this exploratory study, we advocate for further research into other “return to life” experiences—such as dating, parenting, and social media engagement—which are equally vital to recovery as the more commonly measured “return to play” and “return to work” outcomes (49, 57). Gaining insight into how these meaningful aspects of life contribute to emotional well-being, identity reconstruction, and social reintegration can help establish a more holistic framework for recovery. Exploring these areas may also inform the development of interventions that support not only physical healing but also the psychological and social dimensions of rehabilitation.

There are several limitations to this exploratory study. First, the sample included only women who had experienced a concussion, with most participants within the first year of recovery. Because of this, the findings may not be generalizable to men, individuals with moderate to severe TBI, or those further along their brain injury recovery. Future research should investigate whether the prevalence and predictors of PTG differ across gender, TBI severity, and recovery stage. Given the importance of adaptive cognition in PTG (75–77), future research may also benefit from determining the impact of cognitive impairment following varying severity levels of TBI on PTG. Secondly, since participants were recruited online, it is possible that PTG prevalence and its associative factors may differ among offline populations. Additionally, in the absence of a comparison group, the present study cannot determine whether the relationships between PTG and associative factors such as parenthood and return to work are unique to concussion. Therefore, caution is warranted when generalizing these results. Thirdly, the cross-sectional design of the study limits causal inference, as PTG and its potential predictors were assessed at the same point in time. Also, including participants at different time points following concussion introduces heterogeneity in recovery stage and may have influenced the observed associations with PTG. Participants closer to their injury date may have been actively adapting to ongoing symptoms and recovery demands, whereas those further from injury may have had more time to process their experience and develop positive psychological changes. Future longitudinal studies examining PTG across distinct stages of concussion recovery are needed to better understand how post-traumatic growth develops over time. Another limitation of this study is that, although parity was assessed, time since childbirth was not collected. Therefore, we were unable to account for potential postpartum-related factors, including hormonal changes, recovery, and sleep disruption, which may influence symptom reporting and psychosocial outcomes such as PTG. Future studies should assess reproductive factors in greater detail to better understand their potential influence on recovery and PTG following concussion. Lastly, there are other potential factors that impact PTG that were not explored in this study. Factors such as socioeconomic status, level of social support, access to healthcare and cultural identity may contribute to the observed differences in PTG. There is scope to investigate such sociocultural factors in future research.

Despite these limitations, this exploratory study is, to our knowledge, the first to examine factors associated with PTG in a cohort of female concussion patients with persistent post-concussion symptoms (PPCS), recruited through online social support networks. The study has several notable strengths. First, it addresses an important and understudied area by examining positive psychological outcomes, specifically post-traumatic growth, following concussion. Second, by focusing exclusively on women, the study contributes to the growing understanding of sex-specific experiences and recovery following concussion. Third, the inclusion of psychological, behavioral, demographic, and rehabilitation-related variables provides a comprehensive assessment of factors potentially associated with post-traumatic growth. Fourth, the use of validated and widely accepted instruments to assess post-traumatic growth, depression, anxiety, post-traumatic stress symptoms, resilience, and flourishing enhances the rigor and reliability of the findings. Finally, the results have clinical relevance by identifying factors associated with post-traumatic growth that may inform future research and contribute to the development of more personalized rehabilitation approaches for women recovering from concussion. By stratifying PTG scores into high and low levels, our exploratory study sets the stage for future PTG-oriented intervention studies that target management of modifiable factors into personalized concussion recovery plans. Our findings can inform the development of rehabilitation approaches that integrate PTG and other protective factors, supporting recovery of the whole person—physically, psychologically, and socially. For those with low PTG, researchers could evaluate how such interventions improve functional outcomes such as returning to work and quality of life/overall wellbeing metrics such as flourishing. Future research should also aim to incorporate brain and blood biomarkers in longitudinal designs (78, 79) to better understand whether biological factors help explain differences in PTG outcomes.

## Data Availability

Data will be made available by the authors upon reasonable request.
