# Supplementary material for: Alcohol use, vision therapy, children, mood … what predicts post-traumatic growth after concussion in women?
Source: Front Glob Womens Health. 2026 Aug 27;7:1815889. doi: 10.3389/fgwh.2026.1815889 (PMC13558141; doi:10.3389/fgwh.2026.1815889)
Supplement: Supplementary file 1 [file Table1.docx]

Adjusted Logit Model:

```
                                                                          Results: Logit
===================================================================================================================================================================
Model:                                            Logit                                                     Method:                                       MLE
Dependent Variable:                               Low PTG and moderate/high PTG                             Pseudo R-squared:                             0.215
Date:                                             2026-07-22 19:20                                          AIC:                                          135.9966
No. Observations:                                 103                                                       BIC:                                          167.6134
Df Model:                                         11                                                        Log-Likelihood:                               -55.998
Df Residuals:                                     91                                                        LL-Null:                                      -71.350
Converged:                                        1.0000                                                    LLR p-value:                                  0.0012271
No. Iterations:                                   6.0000                                                    Scale:                                        1.0000
-------------------------------------------------------------------------------------------------------------------------------------------------------------------
                                                                                  Coef.  Std.Err.    z    P>|z|   [0.025  0.975] Odds Ratio OR 2.5% OR 97.5%  VIF
-------------------------------------------------------------------------------------------------------------------------------------------------------------------
const                                                                            -2.9132   1.0764 -2.7065 0.0068 -5.0229 -0.8035     0.0543  0.0066   0.4477    nan
-------------------------------------------------------------------------------------------------------------------------------------------------------------------
Return to work? (No)						   1
Return to work? (Yes)                                                             1.0275   0.5181  1.9831 0.0474  0.0120  2.0430     2.7941  1.0121   7.7141 1.1473
-------------------------------------------------------------------------------------------------------------------------------------------------------------------
I currently do not consume alcohol, but I used to before my injury	    	   1
I currently consume alcohol, and I also used to before my injury  	          0.9016   0.8882  1.0151 0.3101 -0.8392  2.6424     2.4635  0.4320  14.0465 1.5240
I currently consume alcohol, but I did not before my injury      	         -0.0625   0.6569 -0.0951 0.9242 -1.3500  1.2251     0.9394  0.2592   3.4044 1.6391
I currently do not consume alcohol, neither before my injury      	          0.9660   0.6338  1.5243 0.1274 -0.2761  2.2082     2.6275  0.7587   9.0996 1.6254
-------------------------------------------------------------------------------------------------------------------------------------------------------------------
Race (White/Caucasian)						   1
Race (African American/Black)                                          	          1.3035   0.6459  2.0181 0.0436  0.0376  2.5694     3.6822  1.0383  13.0584 1.3598
Race (Hispanic/Latino)                                                            0.9445   0.8536  1.1065 0.2685 -0.7285  2.6175     2.5714  0.4826  13.7008 1.3461
Race (Other)                                                                      0.3664   1.3549  0.2705 0.7868 -2.2891  3.0220     1.4426  0.1014  20.5317 1.1024
-------------------------------------------------------------------------------------------------------------------------------------------------------------------
Number of services due to concussion/mTBI (1-2)  	   		   1
Number of services due to concussion/mTBI (0)                                     1.0420   0.6816  1.5288 0.1263 -0.2938  2.3778     2.8349  0.7454  10.7814 1.2680
Number of services due to concussion/mTBI (3-4)                                   1.6796   0.7665  2.1913 0.0284  0.1773  3.1819     5.3636  1.1940  24.0931 1.2971
Number of services due to concussion/mTBI (5+)                                    0.1699   0.8681  0.1957 0.8448 -1.5315  1.8714     1.1852  0.2162   6.4971 2.0822
-------------------------------------------------------------------------------------------------------------------------------------------------------------------
Currently receiving vision therapy due to mTBI 				   1
Currently not receiving vision therapy due to mTBI                                1.1279   0.7974  1.4144 0.1572 -0.4350  2.6908     3.0892  0.6473  14.7440 1.7432
===================================================================================================================================================================
```
