# Supplementary material for: Alcohol use, vision therapy, children, mood … what predicts post-traumatic growth after concussion in women?
Source: Front Glob Womens Health. 2026 Aug 27;7:1815889. doi: 10.3389/fgwh.2026.1815889 (PMC13558141; doi:10.3389/fgwh.2026.1815889)
Supplement: Supplementary file 2 [file Table2.docx]

|  | **Low PTG** | **Moderate to high PTG** | **Total (frequency)** | **P-value** |
| --- | --- | --- | --- | --- |
| Are you receiving physical medicine & rehabilitation service due to your concussion/mTBI? | | | | Chi2 p-value |
| *Yes* | *11 (18.3%)* | *7 (12.5%)* | *18 (15.5%)* | *0.542* |
| *No* | *49 (81.7%)* | *49 (87.5%)* | *98 (84.5%)* |  |
| Total | 60 (100%) | 56 (100%) | 116 (100%) |  |
| Are you receiving psychotherapy service due to your concussion/mTBI? | | | | Chi2 p-value |
| *Yes* | *13 (21.7%)* | *8 (14.3%)* | *21 (18.1%)* | *0.429* |
| *No* | *47 (78.3%)* | *48 (85.7%)* | *95 (81.9%)* |  |
| Total | 60 (100%) | 56 (100%) | 116 (100%) |  |
| Are you receiving social work service(s) due to your concussion/mTBI? | | | | Fisher p-value |
| *Yes* | *3 (5%)* | *3 (5.4%)* | *6 (5.2%)* | *1* |
| *No* | *57 (95%)* | *53 (94.6%)* | *110 (94.8%)* |  |
| Total | 60 (100%) | 56 (100%) | 116 (100%) |  |
| Are you receiving any other service(s) due to your concussion/mTBI? | | | | Chi2 p-value |
| *Yes* | *11 (18.3%)* | *8 (14.3%)* | *19 (16.4%)* | *0.736* |
| *No* | *49 (81.7%)* | *48 (85.7%)* | *97 (83.6%)* |  |
| Total | 60 (100%) | 56 (100%) | 116 (100%) |  |

|  | **Low PTG** | **Moderate to high PTG** | **Total (frequency)** | **P-value** |
| --- | --- | --- | --- | --- |
| **HISTORICAL SUBSTANCE USE** | | | | |
| **Tobacco history** | | | | |
| *Yes (Used to consume tobacco before injury)* | *13 (21.7%)* | *20 (35.7%)* | *33 (28.4%)* | *p: 0.174* |
| *No (Did not consume tobacco before injury)* | *45 (75%)* | *36 (64.3%)* | *81 (69.8%)* |  |
| *Missing* | *2 (3.3%)* | *0 (0%)* | *2 (1.7%)* |  |
| Total | 60 (100%) | 56 (100%) | 116 (100%) |  |
| **Alcohol history** | | | | |
| *Yes (Used to consume alcohol before injury)* | *39 (65%)* | *22 (39.3%)* | *61 (52.6%)* | *p: 0.004* |
| *No (Did not consume alcohol before injury)* | *17 (28.3%)* | *32 (57.1%)* | *49 (42.2%)* |  |
| *Missing* | *4 (6.7%)* | *2 (3.6%)* | *6 (5.2%)* |  |
| Total | 60 (100%) | 56 (100%) | 116 (100%) |  |
| **Marijuana history** | | | | |
| *Yes (Used to consume marijuana before injury)* | *17 (28.3%)* | *8 (14.3%)* | *25 (21.6%)* | *p: 0.16* |
| *No (Did not consume marijuana before injury)* | *41 (68.3%)* | *42 (75%)* | *83 (71.6%)* |  |
| *Missing* | *2 (3.3%)* | *6 (10.7%)* | *8 (6.9%)* |  |
| Total | 60 (100%) | 56 (100%) | 116 (100%) |  |

| **Which of these best describe you?** | | | | **Fisher p-value** |
| --- | --- | --- | --- | --- |
| *I use marijuana for medical reasons* | *6 (10%)* | *7 (12.5%)* | *13 (11.2%)* | 0.5624 |
| *I use marijuana recreationally* | *6 (10%)* | *3 (5.4%)* | *9 (7.8%)* |  |
| *I use marijuana for medical and recreational reasons* | *10 (16.7%)* | *6 (10.7%)* | *16 (13.8%)* |  |
| *None of the above* | *34 (56.7%)* | *37 (66.1%)* | *71 (61.2%)* |  |
| *Missing* | *4 (6.7%)* | *3 (5.4%)* | *7 (6%)* |  |

|  | **Low PTG** | **Moderate to high PTG** | **Total (frequency)** | **P-value** |
| --- | --- | --- | --- | --- |
| **Since your injury, have you been able to return social media (Tik Tok, Facebook, Twitter(X), LinkedIn etc)?** | | | | **Chi2 p-value** |
| *Yes* | *51 (85%)* | *45 (80.4%)* | *96 (82.8%)* | 0.326 |
| *No* | *6 (10%)* | *10 (17.9%)* | *16 (13.8%)* |  |
| *Not Applicable* | *3 (5%)* | *1 (1.8%)* | *4 (3.4%)* |  |
| Total | 60 (100%) | 56 (100%) | 116 (100%) |  |
| **Since your injury, have you been able to return dating/marriage/relationships?** | | | | **Chi2 p-value** |
| *Yes* | *47 (78.3%)* | *42 (75%)* | *89 (76.7%)* | 0.084 |
| *No* | *7 (11.7%)* | *12 (21.4%)* | *19 (16.4%)* |  |
| *Not Applicable* | *6 (10%)* | *1 (1.8%)* | *7 (6%)* |  |
| *Missing* | *0 (0%)* | *1 (1.8%)* | *1 (0.9%)* |  |
| Total | 60 (100%) | 56 (100%) | 116 (100%) |  |
| **Since your injury, have you been able to return to parenting?** | | | | **Chi2 p-value** |
| *Yes* | *22 (36.7%)* | *28 (50%)* | *50 (43.1%)* | 0.237 |
| *No* | *7 (11.7%)* | *7 (12.5%)* | *14 (12.1%)* |  |
| *Not Applicable* | *31 (51.7%)* | *20 (35.7%)* | *51 (44%)* |  |
| *Missing* | *0 (0%)* | *1 (1.8%)* | *1 (0.9%)* |  |
| Total | 60 (100%) | 56 (100%) | 116 (100%) |  |

|  | | count | | mean | | std | | min | | 25% | | 50% | | 75% | | max | |  |
| --- | --- | --- | --- | --- | --- | --- | --- | --- | --- | --- | --- | --- | --- | --- | --- | --- | --- | --- |
| Total resilience score | | | | | | | | | | | | | | | | | | |
| Low | 60 | | 121.0 | | 20.5 | | 80 | | 107 | | 115.5 | | 137.5 | | 161 | | T-statistic: -2.8 P-value: 0.005*** | |
| Moderate to high | 56 | | 132.6 | | 23.8 | | 64 | | 114.8 | | 131.5 | | 153.3 | | 175 | |  |  |
| Total | **116** | | **126.6** | | **22.8** | | **64** | | **110** | | **124** | | **146** | | **175** | |  |  |
| Total flourishing score | | | | | | | | | | | | | | | | | | |
| Low | 60 | | 72.8 | | 18.7 | | 22 | | 60.8 | | 74 | | 86.3 | | 108 | | T-statistic: -2.9 P-value: 0.005*** | |
| Moderate to high | 56 | | 83.1 | | 19.9 | | 42 | | 67 | | 86.5 | | 98.5 | | 119 | |  |  |
| Total | **116** | | **77.8** | | **19.9** | | **22** | | **64.75** | | **78.5** | | **91.2** | | **119** | |  |  |
