# Supplementary material for: Alcohol use, vision therapy, children, mood … what predicts post-traumatic growth after concussion in women?
Source: Front Glob Womens Health. 2026 Aug 27;7:1815889. doi: 10.3389/fgwh.2026.1815889 (PMC13558141; doi:10.3389/fgwh.2026.1815889)
Supplement: Supplementary file 3 [file Table3.docx]

Adjusted OLS model:

| OLS Regression Results  Dep. Variable: Total PTG score R-squared: 0.280  Model: OLS Adj. R-squared: 0.193  Method: Least Squares F-statistic: 3.216  Date: Fri, 17 Jul 2026 Prob (F-statistic): 0.000986  Time: 16:56:55 Log-Likelihood: -467.80  No. Observations: 103 AIC: 959.6  Df Residuals: 91 BIC: 991.2  Df Model: 11  Covariance Type: nonrobust | | | | | | | |
| --- | --- | --- | --- | --- | --- | --- | --- |
|  | Coef. | Std.Err. | t | P>\|t\| | [0.025 | 0.975] | VIF |
| const | 44.0944 | 10.1838 | 4.3298 | 0 | 23.8655 | 64.3233 |  |
| Return to work? (No) | 1 |  |  |  |  |  |  |
| Return to work? (Yes) | 10.5944 | 5.2318 | 2.025 | 0.0458 | 0.202 | 20.9867 | 1.1473 |
| I currently do not consume alcohol, but I used to before my injury | 1 |  |  |  |  |  |  |
| I currently consume alcohol, and I also used to before my injury | 4.8422 | 9.1602 | 0.5286 | 0.5984 | -13.3534 | 23.0378 | 1.524 |
| I currently consume alcohol, but I did not before my injury | -5.5953 | 6.9299 | -0.8074 | 0.4215 | -19.3607 | 8.1701 | 1.6391 |
| I currently do not consume alcohol, neither before my injury | 12.6775 | 6.454 | 1.9643 | 0.0525 | -0.1426 | 25.4976 | 1.6254 |
| Race (White/Caucasian) | 1 |  |  |  |  |  |  |
| Race (African American/Black) | 13.3522 | 6.5666 | 2.0334 | 0.0449 | 0.3085 | 26.3959 | 1.3598 |
| Race (Hispanic/Latino) | 5.4834 | 8.9427 | 0.6132 | 0.5413 | -12.2801 | 23.2469 | 1.3461 |
| Race (Other) | -8.4959 | 14.8639 | -0.5716 | 0.569 | -38.0213 | 21.0295 | 1.1024 |
| Number of services received due to concussion/mTBI (1-2) | 1 |  |  |  |  |  |  |
| Number of services received due to concussion/mTBI (0) | 12.2161 | 7.2212 | 1.6917 | 0.0941 | -2.1278 | 26.5601 | 1.268 |
| Number of services received due to concussion/mTBI (3-4) | 12.1488 | 6.9903 | 1.738 | 0.0856 | -1.7365 | 26.0342 | 1.2971 |
| Number of services received due to concussion/mTBI (5+) | 2.9038 | 8.8565 | 0.3279 | 0.7438 | -14.6885 | 20.4962 | 2.0822 |
| Currently receiving vision therapy due to mTBI | 1 |  |  |  |  |  |  |
| Currently not receiving vision therapy due to mTBI | 9.8531 | 7.9457 | 1.2401 | 0.2181 | -5.9301 | 25.6363 | 1.7432 |

Crude OLS Models

a) Race

```
                            OLS Regression Results
==============================================================================
Dep. Variable:        Total PTG score   R-squared:                       0.136
Model:                            OLS   Adj. R-squared:                  0.110
Method:                 Least Squares   F-statistic:                     5.186
Date:                Wed, 22 Jul 2026   Prob (F-statistic):            0.00228
Time:                        19:24:50   Log-Likelihood:                -477.19
No. Observations:                 103   AIC:                             962.4
Df Residuals:                      99   BIC:                             972.9
Df Model:                           3
Covariance Type:            nonrobust
============================================================================================================
                                               coef    std err          t      P>|t|      [0.025      0.975]
------------------------------------------------------------------------------------------------------------
const                                       64.6000      3.147     20.524      0.000      58.355      70.845
------------------------------------------------------------------------------------------------------------
Race (White/Caucasian)			          1
Race (African American/Black)    	    23.0667      6.061      3.806      0.000      11.040      35.093
Race (Hispanic/Latino)            	     2.8545      8.273      0.345      0.731     -13.561      19.270
Race (Other)                     	    -8.2667     14.985     -0.552      0.582     -38.000      21.467
==============================================================================
Omnibus:                        0.014   Durbin-Watson:                   2.198
Prob(Omnibus):                  0.993   Jarque-Bera (JB):                0.135
Skew:                          -0.012   Prob(JB):                        0.935
Kurtosis:                       2.824   Cond. No.                         6.24
==============================================================================
```

b) Return to work

```
                            OLS Regression Results
==============================================================================
Dep. Variable:        Total PTG score   R-squared:                       0.045
Model:                            OLS   Adj. R-squared:                  0.035
Method:                 Least Squares   F-statistic:                     4.709
Date:                Wed, 22 Jul 2026   Prob (F-statistic):             0.0323
Time:                        19:28:36   Log-Likelihood:                -482.36
No. Observations:                 103   AIC:                             968.7
Df Residuals:                     101   BIC:                             974.0
Df Model:                           1
Covariance Type:            nonrobust
===============================================================================================
                                  coef    std err          t      P>|t|      [0.025      0.975]
-----------------------------------------------------------------------------------------------
const                          62.9500      4.177     15.071      0.000      54.664      71.236
-----------------------------------------------------------------------------------------------
Return to work? (No)		       1
Return to work? (Yes)	        11.5897      5.341      2.170      0.032       0.995      22.184
==============================================================================
Omnibus:                        2.665   Durbin-Watson:                   2.114
Prob(Omnibus):                  0.264   Jarque-Bera (JB):                2.673
Skew:                          -0.373   Prob(JB):                        0.263
Kurtosis:                       2.741   Cond. No.                         2.97
==============================================================================
```

c) Alcohol use

```
                            OLS Regression Results
==============================================================================
Dep. Variable:        Total PTG score   R-squared:                       0.102
Model:                            OLS   Adj. R-squared:                  0.075
Method:                 Least Squares   F-statistic:                     3.764
Date:                Wed, 22 Jul 2026   Prob (F-statistic):             0.0132
Time:                        19:35:24   Log-Likelihood:                -479.15
No. Observations:                 103   AIC:                             966.3
Df Residuals:                      99   BIC:                             976.8
Df Model:                           3
Covariance Type:            nonrobust
====================================================================================================================================================
                                                                                       coef    std err          t      P>|t|      [0.025      0.975]
----------------------------------------------------------------------------------------------------------------------------------------------------
const                                                                               64.2667      4.722     13.611      0.000      54.898      73.635
----------------------------------------------------------------------------------------------------------------------------------------------------
I currently do not consule alcohol, but I used to before my injury		   1
I currently consume alcohol, and I also used to before my injury    	        10.6500      8.833      1.206      0.231      -6.877      28.177
I currently consume alcohol, but I did not before my injury                         -3.5259      6.860     -0.514      0.608     -17.138      10.087
I currently do not consume alcohol, neither before my injury                	        16.5275      6.478      2.551      0.012       3.674      29.381
==============================================================================
Omnibus:                        0.555   Durbin-Watson:                   2.220
Prob(Omnibus):                  0.758   Jarque-Bera (JB):                0.539
Skew:                          -0.169   Prob(JB):                        0.764
Kurtosis:                       2.895   Cond. No.                         4.71
==============================================================================
```

d) Number of services received due to concussion/mTBI

```
                            OLS Regression Results
==============================================================================
Dep. Variable:        Total PTG score   R-squared:                       0.065
Model:                            OLS   Adj. R-squared:                  0.037
Method:                 Least Squares   F-statistic:                     2.295
Date:                Wed, 22 Jul 2026   Prob (F-statistic):             0.0825
Time:                        19:42:05   Log-Likelihood:                -481.25
No. Observations:                 103   AIC:                             970.5
Df Residuals:                      99   BIC:                             981.0
Df Model:                           3
Covariance Type:            nonrobust
=========================================================================================================================
                                                            coef    std err          t      P>|t|      [0.025      0.975]
-------------------------------------------------------------------------------------------------------------------------
const                                                    69.0833      3.810     18.134      0.000      61.524      76.643
-------------------------------------------------------------------------------------------------------------------------
Number of services due to concussion/mTBI (1-2) 	        1
Number of services due to concussion/mTBI (0)       	   7.6814      7.449      1.031      0.305      -7.100      22.463
Number of services due to concussion/mTBI (3-4)     	   9.2325      7.154      1.291      0.200      -4.963      23.428
Number of services due to concussion/mTBI (5+)    	 -10.9254      7.154     -1.527      0.130     -25.121       3.270
==============================================================================
Omnibus:                        1.618   Durbin-Watson:                   2.083
Prob(Omnibus):                  0.445   Jarque-Bera (JB):                1.508
Skew:                          -0.293   Prob(JB):                        0.471
Kurtosis:                       2.908   Cond. No.                         3.83
==============================================================================
```

e) Currently receiving vision therapy

```
                            OLS Regression Results
==============================================================================
Dep. Variable:        Total PTG score   R-squared:                       0.069
Model:                            OLS   Adj. R-squared:                  0.060
Method:                 Least Squares   F-statistic:                     7.506
Date:                Wed, 22 Jul 2026   Prob (F-statistic):            0.00727
Time:                        19:44:44   Log-Likelihood:                -481.02
No. Observations:                 103   AIC:                             966.0
Df Residuals:                     101   BIC:                             971.3
Df Model:                           1
Covariance Type:            nonrobust
============================================================================================================================
                                                               coef    std err          t      P>|t|      [0.025      0.975]
----------------------------------------------------------------------------------------------------------------------------
const                                                       55.7000      5.830      9.554      0.000      44.134      67.266
----------------------------------------------------------------------------------------------------------------------------
Currently receiving vision therapy due to mTBI    		 1
Currently not receiving vision therapy due to mTBI          17.7940      6.495      2.740      0.007       4.910      30.678
==============================================================================
Omnibus:                        1.296   Durbin-Watson:                   2.178
Prob(Omnibus):                  0.523   Jarque-Bera (JB):                1.211
Skew:                          -0.261   Prob(JB):                        0.546
Kurtosis:                       2.903   Cond. No.                         4.33
==============================================================================
```
